# Overweight and Obese Adult Patients Show Larger Benefits from Concurrent Training Compared with Pharmacological Metformin Treatment on Insulin Resistance and Fat Oxidation

**DOI:** 10.3390/ijerph192114331

**Published:** 2022-11-02

**Authors:** Jairo Azócar-Gallardo, Rodrigo Ramirez-Campillo, José Afonso, Mário Sá, Urs Granacher, Luis González-Rojas, Alex Ojeda-Aravena, José Manuel García-García

**Affiliations:** 1 Programa de Investigación en Deporte, Sociedad y Buen Vivir (DSBv), Universidad de Los Lagos, Osorno 5290000, Chile; 2 Departamento de Ciencias de la Actividad Física, Universidad de Los Lagos, Puerto Montt 5480000, Chile; 3 Facultad de Ciencias del Deporte, Universidad de Castilla-La Mancha (UCLM), 45071 Toledo, Spain; 4 Exercise and Rehabilitation Sciences Institute, School of Physical Therapy, Faculty of Rehabilitation Sciences, Universidad Andres Bello, Santiago 7591538, Chile; 5 Centre for Research, Education, Innovation and Intervention in Sport (CIFI2D), Faculty of Sport, University of Porto, 4200-450 Porto, Portugal; 6 Faculdade de Motricidade Humana, Universidade de Lisboa, 1495-751 Lisboa, Portugal; 7 Department of Sport and Sport Science, Exercise and Human Movement Science, University of Freiburg, 79102 Freiburg, Germany; 8 Centro Tratamiento de la Obesidad, Pontificia Universidad Católica de Chile, Santiago 7550000, Chile; 9 IRyS Group, Physical Education School, Pontificia Universidad Católica de Valparaíso, Valparaíso 2581967, Chile

**Keywords:** resistance training, endurance training, muscle strength, human physical conditioning, musculoskeletal and neural physiological phenomena, exercise

## Abstract

Metformin, a drug widely used to treat insulin resistance, and training that combines aerobic and strength exercise modalities (i.e., concurrent training) may improve insulin sensitivity. However, there is a paucity of clinical trials investigating the effects of concurrent training, particularly on insulin resistance and fat oxidation in overweight and obese patients. Furthermore, only a few studies have compared the effects of concurrent training with metformin treatment. Therefore, the aim of this study was to examine the effects of a 12-week concurrent training program versus pharmaceutical treatment with metformin on maximum fat oxidation, glucose metabolism, and insulin resistance in overweight or obese adult patients. Male and female patients with insulin resistance were allocated by convenience to a concurrent training group (*n* = 7 (2 males); age = 32.9 ± 8.3 years; body mass index = 30 ± 4.0 kg·m^−2^) or a metformin group (*n* = 7 (2 males); age = 34.4 ± 14.0 years; body mass index = 34.4 ± 6.0 kg·m^−2^). Before and after the interventions, all participants were assessed for total body mass, body mass index, fat mass, fat-free mass, maximum oxygen consumption, maximal fat oxidization during exercise, fasting glucose, and insulin resistance through the homeostatic model assessment (HOMA-IR). Due to non-normal distribution of the variable maximal fat oxidation, the Mann–Whitney U test was applied and revealed better maximal fat oxidization (Δ = 308%) in the exercise compared with the metformin group (Δ = −30.3%; *p* = 0.035). All other outcome variables were normally distributed, and significant group-by-time interactions were found for HOMA-IR (*p* < 0.001, Δ = −84.5%), fasting insulin (*p* < 0.001, Δ = −84.6%), and increased maximum oxygen consumption (*p* = 0.046, Δ = 12.3%) in favor of the exercise group. Similar changes were found in both groups for the remaining dependent variables. Concurrent training seems to be more effective compared with pharmaceutical metformin treatment to improve insulin resistance and fat oxidation in overweight and obese adult patients with insulin resistance. The rather small sample size calls for more research in this area.

## 1. Introduction

Worldwide, diabetes is the leading cause of blindness, non-traumatic lower-extremity amputations, peripheral neuropathy, and end-stage renal disease, and is a major risk factor for atherosclerotic cardiovascular disease and mortality, accounting for 43% of all diabetes-related deaths in people under 70 years of age [1]. Insulin resistance (IR) is a disorder of glucose homeostasis, involving reduced insulin sensitivity (IS) in muscle, adipose tissue, liver, and other tissues where insulin action is involved [2]. Insulin resistance is considered the best predictor [3] and main etiological factor in the development of type 2 diabetes [4,5].

Physical inactivity and a poor diet (e.g., excessive content of fat and carbohydrates) have been associated with an increased risk of developing IR [6]. One of the characteristics of IR is attributed to metabolic inflexibility, a concept related to the poor utilization of glucose and fatty acids [6,7]. Kelley et al. (1999) reported that with obesity, IR derives not only from the absorption of fatty acids, but also from the metabolic inflexibility for fat oxidation, which favors the accumulation of triglycerides in the skeletal muscle [7]. Indeed, the maximum fat oxidation and the exercise intensity associated with maximum fat oxidation are lower in obese patients compared to individuals with normal body mass [8].

Skeletal muscle therefore plays an integral role in the improvement of IS in patients with IR, and the effective modulation of glucose and fatty acid metabolism in skeletal muscle through exercise or certain pharmacological treatments has been associated with the reversal of IR and the improvement of complications associated with diabetes, such as inflammation and oxidative stress [6]. Metformin, a drug widely used to treat IR [9], and physical exercise may both improve IS [10]. Metformin operates by decreasing hepatic glucose production and increasing hepatic and whole-body fat oxidation [11,12,13]. Metformin also improves glucose utilization and fatty acid oxidation in skeletal muscle [12], which is deregulated in overweight or obese patients with IR, leading to improved IS [14]. However, as to the pharmaceutical treatment of metformin, side effects such as lactic acidosis, dizziness, muscle pain, tiredness, and gastrointestinal intolerance have been reported [15].

Besides pharmaceutical treatment, physical exercise may improve IS in normal and IR populations through multiple adaptations in glucose transport and metabolism [16]. Three training modalities are commonly prescribed for the treatment of IS: aerobic training (e.g., walking, running, cycling), resistance or strength training (e.g., machine-based training or lifting free weights), and combined aerobic and strength training [17]. The performance of physical exercise aimed at developing both aerobic capacity and muscle strength in the same training session, or in different sessions sequenced on the same training day or the same training week, is commonly referred to as concurrent training (CT) [18]. A review by Johannsen et al. (2016) found that CT has the largest effect compared with single-mode training (i.e., either strength or aerobic training) on glucose homeostasis (glycosylated hemoglobin), cardiorespiratory fitness, muscular strength, and abdominal fat reduction in people at risk of or living with type 2 diabetes [19]. The combination of both training modes (i.e., CT) improves oxygen uptake, transport, and utilization, as well as fat oxidation through aerobic exercise [20]. Concomitantly, strength training improves IS through muscle hypertrophy, increased glucose storage and a reduced insulin dosage required to maintain normal glucose tolerance [21]. However, there is a paucity of clinical trials investigating the effects of CT [19], particularly on IR and fat oxidation in overweight and obese patients. Furthermore, only a few studies [22,23] have compared the effects of CT against pharmacological therapy (i.e., metformin).

Therefore, the aim of this study was to examine the effects of a 12-week CT program versus pharmaceutical treatment with metformin on maximum fat oxidation, glucose metabolism, and IR in overweight or obese adult patients with IR. Based on the results from previous studies [19,20,23,24,25], we hypothesized that, compared to metformin treatment, CT would induce larger improvements in maximum fat oxidation and IR in overweight/obese individuals with IR.

## 2. Materials and Methods

### 2.1. Participants

To calculate the required sample size, freeware statistical software (G * Power; University of Düsseldorf, Düsseldorf, Germany) was used. The following variables were included in the a priori power analysis: study design—two groups, test, retest; effect size of 0.40 for the main outcome (i.e., homeostatic model assessment for insulin resistance—HOMA-IR) [22]; alpha error < 0.05; non-sphericity correction = 1; correlation between repeated measures = 0.5; desired power (1-ß error) = 0.80. The results of the a priori power analysis indicated that a minimum of six participants would be needed for each group to achieve statistical significance for HOMA-IR. A larger number of participants were recruited from an obesity treatment center due to potential attrition. The study was conducted at the center, and the study procedures were in accordance with the latest version of the Helsinki Declaration of Ethical Principles for Medical Research Involving Human Subjects. Although we did not pre-register the study protocol, this was submitted to the local ethics committee, approved, and can be requested from the corresponding author. The local ethics committee approved the study protocol (registration number 151007005). This protocol can be made available upon reasonable request.

To be eligible for inclusion, the participants had to: (i) be either overweight (body mass index ≥ 25.0–29.9 kg·m^−2^) or obese (body mass index ≥ 30.0 kg·m^−2^) with IR (HOMA-IR ≥ 2.5–5.0) [26,27]; (ii) be physically inactive, defined as failure to meet the World Health Organization minimum physical activity recommendations for adults (i.e., moderate aerobic physical activity for at least 150 to 300 min per week, or vigorous aerobic physical activity for at least 75 to 150 min per week, or an equivalent combination of moderate and vigorous activity throughout the week) [28]. Exclusion criteria were as follows: (i) HOMA-IR > 5.0 [27]; (ii) consumption of medication (other than metformin) [13].

**Figure 1:**
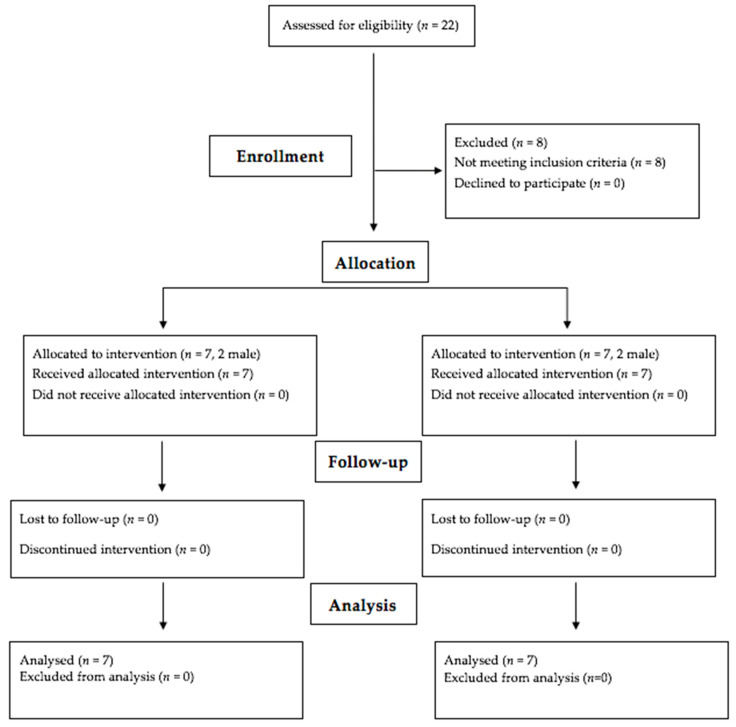
Flow diagram depicting participants’ eligibility and progression during the different study phases.

According to these criteria, 14 participants, including 4 males and 10 females, were eligible to be included in this study. Due to logistical restrictions, randomization of study participants was precluded (i.e., participants in the metformin group were reluctant to exercise). Therefore, the participants were non-randomly allocated into two different experimental groups. Group one (*n* = 7 (2 males/5 females); 1 overweight and 6 obese participants) followed a conventional pharmacological treatment involving metformin (two daily doses of 850 mg, for 12 weeks), maintaining their habitual lifestyle (e.g., physically inactive). The CT group (*n* = 7 (2 males/5 females); 3 overweight and 4 obese participants) completed a 12-week CT program, without pharmaceutical treatment (metformin). A flow diagram of the study progress through the different phases of the trial is depicted in Figure 1. Group-specific descriptive characteristics of the participants are shown in Table 1.

### 2.2. Experimental Design

**Figure 2:**
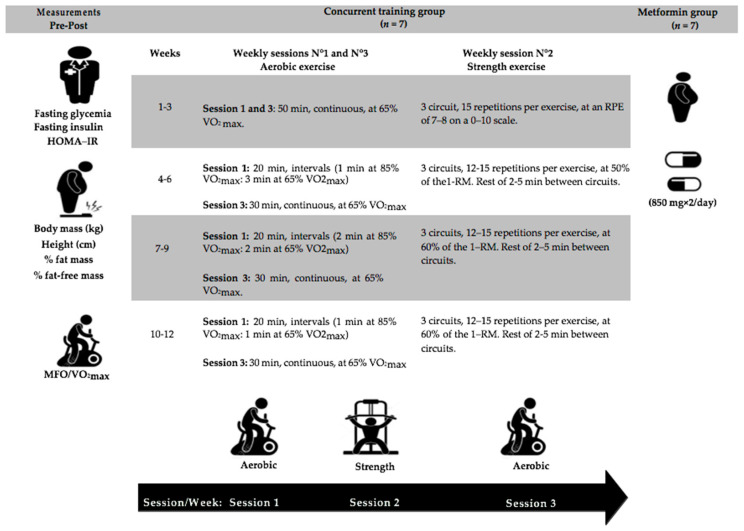
Study design and intervention programs. HOMA-IR: insulin resistance index. VO_2_max: maximum oxygen consumption. MFO: maximal fat oxidation. HR: heart rate. RPE: rating of perceived exertion. 1-RM: one repetition maximum. The intensity of aerobic exercise was prescribed based on the heart rate attained at 65% VO_2_max and 85% of VO_2_max assessed before the intervention.

One week before and one week immediately after the 12-week CT intervention period, fasting glucose and insulin were assessed for the calculation of HOMA-IR, body composition, maximum fat oxidation, and maximal oxygen uptake (VO_2_max) of the participants (Figure 2). Measurements were performed over two days, with fasting glucose and insulin and body composition on the first day, and maximum fat oxidation and VO_2_max on the second day. The principal investigator was blinded during assessment for group allocation. The physician in charge of the patients performed fasting glucose and insulin measurements, while the body composition and metabolic tests (maximum fat oxidation and VO_2_max) were performed by the physiotherapist in charge of performing these types of tests in the treatment center. All test sessions were performed under similar environmental conditions (21–23 °C temperature), at the same time of day, between 8:00 and 10:00 am. To minimize the effects of the circadian rhythm on body composition, fasting glucose and insulin, maximum fat oxidation, and VO_2_max assessments were performed in the same order and at the same time of day [29].

### 2.3. Measurements

#### 2.3.1. Body Composition

Participants attended the laboratory after >6 h and <12 h of fasting. None of the female participants attended the assessments during the menstrual phase. Body height and mass were measured with a stadiometer and digital scale (SECA 217, Hamburg, Germany; accuracy 0.5 cm and 0.1 kg, respectively). Both evaluations were performed without shoes and with light clothing. Body lean and fat mass were assessed with a validated bioimpedance system (Inbody 720, Seoul, Korea) with tetrapolar multifrequency (8 tactile points) [12,30].

#### 2.3.2. Insulin Sensitivity

Insulin sensitivity was assessed through the HOMA-IR [27]. The HOMA-IR was calculated as fasting insulin × fasting glucose/405 [12,31]. The HOMA-IR is a validated method (by euglycemic-hyperinsulinemic clamp), which has been considered the gold standard for IS assessment [12,32], and is often applied in clinical diagnosis for IR and diabetes mellitus type 2 [33]. Based on a previous study conducted with participants from the same country as in this study [34], a HOMA-IR score of 2.5–5.0 was considered to classify participants with IR.

#### 2.3.3. Cardiorespiratory Fitness

Participants attended the laboratory >6 h and <12 h after their last meal and 24 h free from any alcoholic beverage, coffee, drug intake (including metformin), or any other stimulant substance consumption. An incremental test was performed on a cycle ergometer (Technogym Bike Med, Technogym, Gambettola, Italy), adapted from previous recommendations [35]. The theoretical maximum load (W) was estimated [36]. The protocol consisted of a 3 min rest period, and then a 3 min warm-up at 20% of maximal load, followed by 6 min stages at 30, 40, 50, and 60% of maximal load until a respiratory exchange ratio ≥1 was reached. Thereafter, 6 min stages were completed until the maximal effort was reached. Verbal stimuli were allowed. The test was considered maximum if a respiratory exchange ratio ≥ 1.1 was reached and/or if the maximum heart rate (HR_max_) was greater than or equal to the theoretical maximum predicted by the Morris equation for the cycle ergometer test [37]. Considering the last completed cycle ergometer stage, and according to the manufacturer specifications, the following variables were calculated from the average of the last 30 s of exhaled air using the breath-by-breath gas analysis method (Metalyzer 3B-R2. Cortex^®^, Leipzig, Germany): ventilatory threshold 2 (value provided by the MetaSoft^®^ Studio software (Cortex^®^, Leipzig, Germany), and validated via visual inspection), HR_max_ (beats·min^−1^), maximum load (watts). The same methodology was used to determine VO_2_max (L·min^−1^). The evaluators were unaware of the participants’ assignment. Before the test, the patients were educated and familiarized with the test.

#### 2.3.4. Maximal Fat Oxidation

The maximum fat oxidation rate (g × h^−1^) was measured during exercise as previously described for the incremental cycle ergometer test, using the equations of Frayn [38], with the average value of the oxygen and carbon dioxide volumes during the last 2 min of each completed 6 min stage.

#### 2.3.5. Concurrent Training

The CT program lasted 12 weeks, with three weekly exercise sessions, and consisted of a combination of aerobic (cardiorespiratory) exercise on a cycle ergometer and strength training. The first and third session of the week involved aerobic exercises, and the second weekly session involved strength exercises (Figure 2). Each session lasted 60–75 min and was supervised by an expert physiotherapist. All sessions started with a ~15 min warm-up, involving 5 min of cycle ergometer exercise at <65% of VO_2_max, and joint mobility and dynamic stretching. A graphical depiction of the training program is shown in Figure 2.

Aerobic exercise was performed at 65–85% of VO_2_max (assessed before the intervention). During the training sessions, the heart rate was controlled by telemetry (Polar T31, Polar, Kempele, Finland). Of note, the intensity of aerobic exercise was prescribed based on the heart rate attained at 65% of VO_2_max and 85% of VO_2_max assessed before the intervention. Therefore, the progressive overload during the 12 weeks of intervention was based on the exercise intensity, according to the inter-individual adaptive response to the training stimulus.

The participants completed strength exercises for the upper body (chest press, latissimus pull-down, bicep curl) and lower body (leg press, prone femoral curl, leg extension), similar to those performed in other studies [22,25]. During each strength training session, the six strength exercises were completed in a circuit style, with one upper-body exercise performed, followed (after 30–60 s of rest) by a lower-body exercise, until the completion of the six strength exercises (i.e., one lap). The circuit was completed three times per session. During the first three training weeks, the rate of perceived exertion (0–10 scale) was used to control the intensity during strength training (i.e., the target zone was 7–8 on the rate of perceived exertion scale) [39]. The one-repetition maximum was assessed as previously described [40,41] after week 3, week 6, and week 9 to adjust the loads of the strength exercises and to prescribe strength exercise intensity as a percentage of one-repetition maximum (target zone: 50–60%).

#### 2.3.6. Statistical Analyses

Data are presented as means and standard deviations, with a 95% confidence interval. Normal distribution of data was tested and confirmed for most variables (except maximum fat oxidation) using the Shapiro–Wilk test. The Levene test was applied for the assessment of homoscedasticity. To establish the effects of the intervention programs on the dependent variables, a two (group: metformin and training) × two (time: pre, post) repeated measures mixed ANOVA (i.e., within- and between-interaction) was determined for each parameter. When group × time interactions reached the level of significance (i.e., significant F value), Bonferroni adjusted post hoc tests were computed. In case of significant between-group baseline differences, an analysis of covariance was calculated (ANCOVA) with the respective baseline values as covariates. Effect sizes (ES) for the main effects of ‘group’ and ‘time’ as well as group × time interactions were taken from the ANOVA output (partial eta squared (η_p_^2^)). For maximal fat oxidation (non-normal distribution of data), data were described as median and interquartile ranges (IQR, i.e., 25–75%), and the Mann–Whitney U test was used to make between-group comparisons. The alpha level was set at *p* < 0.05. All data analyses were performed with the statistical package (StatSoft 8.0, Tulsa, OK, USA).

## 3. Results

All participants received assigned treatment conditions. Before the intervention began, no significant baseline differences were observed between the CT and metformin groups for any of the dependent variables (Table 2).

CT participants attended all training sessions, and no injuries or adverse events were reported. Participants in the metformin group complied with the prescribed pharmacological treatment, and no adverse effects were recorded.

Before the intervention began, the maximum rate of fat oxidation (g·h^−1^) was 4 (IQR = 0–11.5) and 2.5 (IQR = 0.5–6.5) for the metformin and CT groups, respectively. After the intervention was completed, the maximum rate of fat oxidation (g·h^−1^) was 7 (IQR = 2–10.5) and 11.5 (IQR = 9.5–16.0) for the groups of metformin and CT, respectively. The Mann–Whitney U test revealed a higher maximal fat oxidation for the CT group compared to the metformin group at completion (Δ 308.1% vs. −30.3%; *p* = 0.035). Significant group by time interactions were observed for HOMA-IR (*p* < 0.001, ES = 0.78), fasting insulin (*p* < 0.001, ES = 0.76), VO_2_max (*p* = 0.025, ES = 0.35), and fat mass (*p* < 0.016; ES = 0.39). Almost significant group by time interactions were observed for body mass (*p* = 0.08, ES = 0.23) and body mass index (*p* = 0.054, ES = 0.27). For all interactions, post hoc tests revealed a favorable effect for CT. No other significant group by time interactions were identified.

## 4. Discussion

The objective of this study was to compare the effects of a 12-week CT program versus pharmaceutical metformin treatment on maximum fat oxidation and IS in overweight or obese participants with IR. The CT was more effective than metformin in improving maximum fat oxidation during exercise and IS.

Our results revealed that maximum fat oxidation improved in the CT group compared to the metformin group (*p* < 0.05). This finding contrasts with the results of a previous study conducted in sedentary subjects with type 2 diabetes, where the respiratory exchange ratio was acutely reduced during exercise [42]. However, in line with our findings, exercise induced favorable effects compared to metformin in healthy active subjects [13] and in sedentary IR subjects [24,25]. Indeed, metformin, when combined with exercise, might blunt the effect of exercise therapy on maximum fat oxidation [13,24,25]. An improvement of maximum fat oxidation may help to reverse the metabolic inflexibility for the oxidation of certain fatty acids that accumulate in skeletal muscle cells (e.g., triglycerides, diacylglycerol, and ceramides), described as mediators of IR in overweight or obese patients [43,44]. Indeed, our data indicate an improvement of IS (i.e., HOMA-IR) in the CT group compared to the metformin group (*p* < 0.001). This finding corroborates the previously reported benefits of exercise on IS (up to 30%) compared to metformin [22] in overweight or obese patients with IR. Additionally, our results indicate that IS improvement is mainly reflected by reduced fasting insulin values (*p* < 0.001 vs. metformin group) instead of fasting glycaemia values (*p* = 0.904 vs. metformin group).

Improvements in maximum fat oxidation and IS after CT compared to metformin were in line with the greater improvement in VO_2_max after CT. Benefits derived from CT on IS may be related to increased oxygen uptake, transport, and utilization [20]. This, in turn, improves fat oxidation capacity [20], which, together with proper manipulation of nutrient intake, improves the overweight or obese phenotype [43,45]. Our findings are in line with those of Cadeddu et al. (2014) in patients with IR, where 12 weeks of exercise training induced a greater (*p* < 0.01) improvement in VO_2_max compared to metformin [5]. In contrast, metformin may induce some unwanted side effects. For example, after 16 weeks of metformin use plus aerobic exercise, although hyperglycemic individuals at high cardiovascular risk reduced IR, they had blunted improvements in VO_2_max compared to the exercise-only group [46]. Moreover, metformin alone can decrease cardiorespiratory capacity up to 50% [5,10], with a potentially negative impact on fat oxidation (i.e., −30.3% in our study), and thus also on IS [22].

Consistent with the results discussed above (e.g., increased maximum fat oxidation; increased VO_2_max; increased HOMA-IR), CT induced greater benefits compared to metformin on body mass, body mass index, and fat mass (*p* = 0.016, *p* = 0.08, *p* = 0.054, respectively). Some CT interventions reduced anthropometric scores in overweight and obese patients [47,48]. A randomized trial by Willis et al. (2012) in sedentary (exercise ≤ 1–2 times/week) overweight or moderately obese (body mass index 25–35 kg/m^2^) adults (18–70 years) showed that 8 months of CT reduced total body mass and fat mass more than strength training alone (*p* < 0.05) [48]. On the other hand, it has been reported that metformin can reduce visceral [49], abdominal, and total body fat [12]. These benefits are obtained through the drug’s mechanism of action by decreasing hepatic glucose production, favoring and increasing hepatic and whole-body fat oxidation [49]. However, there is evidence that metformin has opposite effects on fat oxidation during and after an endurance exercise session [13], and that a short-term treatment with metformin (7–9 days with a final dose of 2 g/day) reduces maximal aerobic capacity (VO_2_peak) [50] in healthy subjects. Boule et al., in 2013, reported that increases in VO_2_peak (mL kg ^−^¹ min ^−1^) were approximately twofold greater in non-metformin users compared to metformin users in the endurance or combined training groups [23]. These results could partly explain why, in our study, CT achieves better results in body mass, body mass index, and fat mass. However, given the scarce evidence in the literature on the effects of CT versus pharmaceutical treatment on the improvement of anthropometric variables (e.g., body mass, body mass index, and fat mass), more studies are needed in the future that examine the effects of CT versus pharmaceutical treatment. Finally, regarding fat-free mass, we found no significant changes for either group (i.e., CT or metformin). Skeletal muscle tissue is the most important component of fat-free mass and plays a key role in overall metabolic health [51]. The importance of skeletal muscle lies in the fact that it is responsible for more than 80% of insulin-stimulated glucose uptake [52]. Konopka et al. (2019), after 12 weeks of aerobic training without metformin (placebo), found no change in fat-free mass, although a decrease in body mass and fat mass was noted [10], in line with our findings.

Therefore, IR in skeletal muscle has a major impact on whole-body metabolic homeostasis [6]. In this regard, the study by Willis et al. (2012) demonstrated that CT is more effective than single-mode strength training in increasing lean body mass (*p* < 0.05) [48]. Moreover, CT appears to show larger effects compared with single-mode endurance training on body mass and fat mass reduction [19]. This study showed that CT is more effective than pharmacological treatment with metformin in improving maximum fat oxidation and VO_2_max in overweight and obese adult patients. The combination of endurance and strength training sequenced on different training days during the training week appears to be particularly effective to reverse IR in overweight and obese adult patients because strength training increases muscle mass and endurance training aerobic capacity.

### 4.1. Clinical Application

Two aerobic exercise sessions per week, alternated (72–96 h of rest) with one session of strength exercise per week (Figure 2), is effective in improving aerobic capacity, fat oxidation, fasting insulin, and insulin sensitivity (HOMA-IR) in obese or overweight individuals with IR. More specifically, aerobic sessions may involve 20 min (intervals) and up to 30–50 min (continuous). For shorter and more intense aerobic sessions, intervals of 1 to 3 min may be used, at ~85% of VO_2_max, followed by 1 to 3 min at ~65% of VO_2_max. For longer and less intense aerobic sessions, ~65% of VO_2_max may be effective. Strength training sessions may comprise repeated circuits (~3), with 12–15 repetitions per exercise for upper- and lower-body muscle groups, using ~50% of 1-RM, and with 2–5 min of inter-circuit recovery.

### 4.2. Limitations

Some limitations of the study must be acknowledged. First, the small sample size. Although we calculated the necessary sample size a priori, future confirmatory studies with larger samples may be necessary. Second, we included both men and women (*n* = 2; *n* = 5, respectively), similar to previous research [5,22]. However, due to the small number of participants by gender, an analysis based on gender was precluded. Future studies including larger numbers of men and women may be needed to confirm our results in separate cohorts based on sex. Third, due to logistical constraints, diet was not controlled. We attempted to address this issue by asking open-ended questions (in person or via telephone call) to all the participants every three weeks, aimed at determining if they changed diet or physical activity (e.g., running, walking, cycling, strength training) habits. A detailed list of the questions is available upon reasonable request, directed to the corresponding author. Overall, participants indicated no meaningful changes in diet or physical activity. Nonetheless, we did not incorporate formal measurement techniques. Future research may be needed to assess the potential for diet modifications and their impact on current findings. Finally, the reliability of most measurements was hampered by ethical issues (e.g., the ERB did not authorize repeat blood sampling or maximal exercise testing). However, the literature reports acceptable measures of absolute and/or relative reliability for all applied test outcomes.

## 5. Conclusions

Exercise is medicine [53,54,55]. Twelve weeks of CT is more effective than pharmaceutical treatment with metformin in improving insulin resistance and fat oxidation in overweight and obese adult patients with IR. Accordingly, we recommend implementing CT when treating overweight and obese patients.

## Figures and Tables

**Table 1:** Baseline characteristics of the participants.

Variables	Metformin Group	Concurrent Training Group	*p* Values
Sex (*n* = male/*n* = female)	2/5	2/5	
Age (years)	34.4 ± 14.0	32.9 ± 8.3	*p* = 0.802
Body mass (kg)	94.2 ± 13.9	85.3 ± 19.7	*p* = 0.345
Height (cm)	165.8 ± 7.2	165.1 ± 10.5	*p* = 0.901
Body mass index (kg·m^−2^)	34.4 ± 6.0	30.8 ± 4.0	*p* = 0.217
Fat mass (%)	42.1 ± 12.9	35 ± 8.2	*p* = 0.325
Fat-free mass (%)	53.1 ± 9.1	50.1 ± 14.8	*p* = 0.143

No statistically significant differences between group baselines were observed in any of the variables. Variables are shown as mean ± standard deviation values.

**Table 2:** Pre-post changes for metabolic and anthropometric characteristics of the study participants according to group allocation.

					ANOVA Outcomes		
Variables	Metformin Group (*n* = 7)		Concurrent Training Group (*n* = 7)		Time F(1, 12), *p* (η_p_^2^)	Group F(1, 12), *p* (η_p_^2^)	Group × Time F(1, 12), *p* (η_p_^2^)
	Pre	Post (Δ%, 95% CI)	Pre	Post (Δ%, 95% CI)			
HOMA-IR	3.2 ± 1.0	3.2 ± 1.3 (−4.0, −23.0 to 19.8)	3.6 ± 0.7	0.6 ± 0.4 (−84.5, −91.9 to −70.3)	F = 43.5, *p* < 0.001 (0.78)	F = 6.9, *p* = 0.021 (0.36)	F = 43.0, *p* < 0.001 (0.78) *
Fasting glycemia (mg·dL^−1^)	83.4 ± 8.2	82.9 ± 6.9 (−0.6, −6.2 to 5.4)	88.3 ± 4.0	88.0 ± 3.4 (−0.3, −3.6 to −3.1)	F = 0.1, *p* = 0.720 (0.01)	F = 2.9, *p* = 0.116 (0.19)	F = 0.0, *p* = 0.904 (0.00)
Fasting insulin (mg·dL^−1^)	15.6 ± 3.7	15.7 ± 3.7 (−3.4, −24.4 to 23.4)	16.4 ± 3.3	2.9 ± 1.9 (−84.6, −92.0 to −70.7)	F = 38.1, *p* < 0.001 (0.76)	F = 10.9, *p* < 0.001 (0.47)	F = 39.3, *p* < 0.001 (0.76) *
VO_2_max (L·min^–1^)	2.1 ± 0.6	2.1 ± 0.7 (−2.9, −11.3 to 6.4)	2.1 ± 0.9	2.4 ± 1.1 (12.3, 1.3 to 24.5)	F = 3.8, *p* = 0.073 (0.24)	F = 0.2, *p* = 0.698 (0.01)	F = 6.5, *p* = 0.025 (0.35) *
Body mass (kg)	94.2 ± 13.9	89.4 ± 14.8 (−5.4, −8.1 to −2.6)	85.3 ± 19.7	77.2 ± 19.1(−9.7, −13.7 to −5.5)	F = 58.6, *p* < 0.001 (0.83)	F = 1.4, *p* = 0.270 (0.10)	F = 3.6, *p* = 0.08 (0.23) *
BMI (kg·m^−2^)	34.4 ± 6.0	32.6± 6.0(−5.3, −8.0 to −2.6)	30.8 ± 4.0	27.9 ± 3.7 (−9.7, −13.8 to −5.4)	F = 49.1, *p* < 0.001 (0.80)	F = 2.6, *p* = 0.130 (0.17)	F = 4.5, *p* = 0.054 (0.27) *
Fat mass (%)	42.1 ± 12.9	36.3 ± 13.7 (−13.4, −19.0 to −7.4)	35.0 ± 8.2	27.3 ± 6.5 (−22.8, −27.4 to −17.9)	F = 136.8, *p* < 0.001 (0.91)	F = 1.7, *p* = 0.217 (0.12)	F = 7.7, *p* = 0.016 (0.39) *
Fat-free mass (%)	53.1 ± 9.1	53.1 ± 9.7 (−0.1, −2.5 to 2.8)	50.1 ± 14.8	49.9 ± 15.0 (−0.3, −3.2 to −2.7)	F = 0.0, *p* = 0.927 (0.00)	F = 0.2, *p* = 0.651 (0.01)	F = 0.0, *p* = 0.927 (0.00)

Variables are presented as means ± standard deviations. CI: confidence interval; BMI: body mass index; ANOVA: analysis of variance; *: denotes significant (and near to) main effects. HOMA-IR: insulin resistance index. VO_2_max: maximum oxygen consumption. η_p_^2^: partial eta squared effect sizes taken from the ANOVA.

## Data Availability

Data may be requested from the corresponding author.
